# A comparison of the COVID-19 response for urban underserved patients experiencing healthcare transitions in three Canadian cities

**DOI:** 10.17269/s41997-022-00651-7

**Published:** 2022-06-30

**Authors:** Ginetta Salvalaggio, Elaine Hyshka, Cara Brown, Andrew D. Pinto, Gayle Halas, Lee Green, Brynn Kosteniuk, Melissa Perri, Nathaniel Le Chalifoux, Garrett Halas, Liane Steiner, Teresa Cavett, Stephanie Montesanti

**Affiliations:** 1 Department of Family Medicine, University of Alberta, 610 University Terrace, Edmonton, AB T6G 2T4 Canada; 2 School of Public Health, University of Alberta, 3-300 Edmonton Clinic Health Academy, 11405 87 Avenue NW, Edmonton, Alberta T6G 1C9 Canada; 3 Department of Occupational Therapy, University of Manitoba, 771 McDermot Ave, Winnipeg, MB R3E 0T6 Canada; 4 Upstream Lab, MAP/Centre for Urban Health Solutions, Li Ka Shing Knowledge Institute, Unity Health Toronto, 30 Bond Street, Toronto, ON M5B 1W8 Canada; 5 Rady Chair, Interprofessional Collaborative Practice, Rady Faculty of Health Sciences, University of Manitoba, P219-770 Bannatyne Ave., Winnipeg, MB R3E 0W3 Canada; 6 Department of Family Medicine, University of Alberta, 516 University Terrace, Edmonton, AB T6G 2T4 Canada; 7 Dalla Lana School of Public Health, University of Toronto, 155 College Street, Toronto, Ontario M5T 3M7 Canada; 8 Rady Faculty of Health Sciences, University of Manitoba, 770 Bannatyne University of Manitoba, Winnipeg, R3E 0W3 Canada; 9 Department of Family Medicine, University of Manitoba, Northern Connection Medical Centre, 2700 McPhillips St, Winnipeg, MB R2V 3M3 Canada; 10 School of Public Health, University of Alberta, 3-266 Edmonton Clinic Health Academy, 11205-87 Avenue NW, Edmonton, Alberta T6G 1C9 Canada

**Keywords:** Medically underserved population, Transition of care, COVID-19 pandemic, Healthcare policy, Population mal desservie en soins de santé, transition de soin, pandémie COVID-19, politique des soins de santé

## Abstract

**Objectives:**

The COVID-19 pandemic and response has highlighted existing strengths within the system of care for urban underserved populations, but also many fault lines, in particular during care transitions. The objectives of this study were to describe COVID-19 response policies for urban underserved populations in three Canadian cities; examine how these policies impact continuity of care for urban underserved populations; determine whether and how urban underserved community members were engaged in policy processes; and develop policy and operational recommendations for optimizing continuity of care for urban underserved populations during public health crises.

**Methods:**

Using Walt & Gilson’s Policy Triangle framework as a conceptual guide, 237 policy and media documents were retrieved. Five complementary virtual group interview sessions were held with 22 front-line and lived-experience key informants to capture less well-documented policy responses and experiences. Documents and interview transcripts were analyzed inductively for policy content, context, actors, and processes involved in the pandemic response.

**Results:**

Available documents suggest little focus on care continuity for urban underserved populations during the pandemic, despite public health measures having disproportionately negative impacts on their care. Policy responses were largely reactive and temporary, and community members were rarely involved. However, a number of community-based initiatives were developed in response to policy gaps. Promising practices emerged, including examples of new multi-level and multi-sector collaboration.

**Conclusion:**

The pandemic response has exposed inequities for urban underserved populations experiencing care transitions; however, it has also exposed system strengths and opportunities for improvement to inform future policy direction.

## Résumé

**Objectifs:**

La pandémie et la riposte au COVID-19 ont mis en évidence les forces existantes au sein du système de soins pour les populations urbaines mal desservies, mais aussi de nombreuses faillites, en particulier lors des transitions de soins. Les objectifs de cette étude étaient de décrire les politiques de réponse au COVID-19 pour les populations urbaines mal desservies dans trois villes canadiennes; examiner l’impact de ces politiques sur la continuité des soins pour les populations urbaines mal desservies; déterminer si et comment les membres de la communauté urbaine mal desservie ont été impliqués dans les processus politiques; et développer des recommandations politiques et opérationnelles pour optimiser la continuité des soins pour les populations urbaines mal desservies pendant les crises de santé publique.

**Méthodes:**

Utilisant le cadre Policy Triangle de Walt et Gilson comme guide conceptuel, 237 documents politiques et des médias ont été récupérés. Cinq séances d’entrevues de groupe virtuelles complémentaires ont été organisées avec 22 informateurs clés de première ligne et d’expérience vécue pour saisir des réponses et des expériences politiques moins bien documentées. Les documents et les transcriptions des entrevues ont été analysés de manière inductive pour le contenu politique, le contexte, les acteurs et les processus impliqués dans la riposte à la pandémie.

**Résultats:**

Les documents disponibles suggèrent que l’accent est peu mis sur la continuité des soins pour les populations urbaines mal desservies pendant la pandémie, malgré les mesures de santé publique ayant des impacts négatifs disproportionnés sur leurs soins. Les réponses politiques étaient en grande partie réactives et temporaires, et les membres de la communauté étaient rarement impliqués. Cependant, un certain nombre d’initiatives communautaires ont été élaborées en réponse aux lacunes des politiques. Des pratiques prometteuses ont émergé, y compris des exemples de nouvelles collaborations multiniveaux et multisectorielles.

**Conclusion:**

La réponse à la pandémie a révélé des inégalités pour les populations urbaines mal desservies qui subissent des transitions de soins, mais elle a également exposé les forces du système et les possibilités d’amélioration pour éclairer l’orientation future des politiques.

## Introduction

The COVID-19 pandemic and subsequent public health response have compounded pre-existing health and social inequities. Urban structurally vulnerable populations encompass those individuals who face additional risk factors or inequalities related to the social determinants of health, and perpetuated through underlying systemic or structural factors (Clark & Preto, 2018). When these vulnerabilities affect access to quality healthcare, an individual is considered underserved. There are many ways that people can be vulnerable to poor health outcomes and experience inadequate access to health and social care as compared with the general population (Patra et al., 2007; Pottie et al., 2020). For instance, communities living with poverty, unstable housing, and more frequent physical and mental illness and substance use also experience challenges accessing effective care. These proximal determinants of health intersect with more distal influences, such as the impact of racism and Canada’s colonial legacy, to compound medical underservice for urban Indigenous peoples (McLane et al., 2022; Browne et al., 2010). Despite the potential for high-continuity care environments to reduce the morbidity related to social determinants of health, provide safer and higher-quality care, improve trust and satisfaction with care, and lower system costs (Starfield & Shi, 2004), urban structurally vulnerable populations report high unmet need for care (Bhui et al., 2006), often seek acute care as their main access point into the healthcare system (Hwang et al., 2011), face obstacles in accessing primary care (Greysen et al., 2014), and describe being treated poorly when accessing care (Martins, 2008). Predisposing adverse circumstances, fewer enabling supports, and intersecting complex needs create barriers to successful transitions from one care space (e.g. hospital) to another (e.g. shelter-based care) (Gelberg et al., 2000; Martins, 2008; Virapongse & Misky, 2018). Further, the traditional organization of services exposes structurally vulnerable patients to a “candidacy”-oriented negotiation process, in which patients must prove their worth when seeking services (Dixon-Woods et al., 2006). Thus, structurally vulnerable populations are not well managed by the formal health system, and medically underserved.

The arrival of the COVID-19 pandemic to Canada has introduced additional challenges to this medically underserved group. Urban underserved populations are at higher risk of COVID-19 infection due to social factors precluding the ability to effectively engage in physical distancing and/or handwashing (Green et al., 2021; Okonkwo et al., 2021; Tsai & Wilson, 2020). They are more likely to experience severe COVID-19 illness due to comorbidities, and experience exacerbation of baseline poor health due to constrained access to harm reduction and treatment options for conditions such as substance use disorders and/or mental illnesses (Green et al., 2020; Henderson et al., 2021; Karamouzian et al., 2020). Moreover, countermeasures to control COVID-19 transmission have required modified approaches to care, including replacing in-person visits with virtual healthcare, restricting visitor and outreach activities in healthcare facilities, and temporarily closing community clinics and municipal amenities. The unintended consequence of these measures is disruption in the continuum of supports typically available to urban underserved populations. Since they may not have sufficient resources to adapt, the urban underserved may experience disproportionate deterioration in continuity of care despite their higher risk of illness and illness severity (Douglas et al., 2020; Smith & Judd, 2020; Tsai & Wilson, 2020).

Around the globe, healthcare providers, outreach workers, community advocates, and other stakeholders tending to the health of urban underserved populations were called upon to mobilize supports in response to pandemic-related challenges. Research has exposed the increased risks and challenges experienced by individuals dealing with substance use and homelessness during COVID-19 (Aronowitz et al., 2021; Parkes et al., 2021; Pixley et al., 2021). The response to COVID-19 drew in part from experience with epidemics, natural disasters, and other emergencies (Edgington, 2009; Leung et al., 2008), and also from communities’ history of mobilization due to government inaction (Friedman et al., 2007). However, these earlier experiences provide little specific policy guidance on how to optimize care continuity and reduce care fragmentation during a viral pandemic response, thus leading to challenges in addressing unique population needs during the COVID-19 pandemic.

## Objectives

The COVID-19 pandemic and response has highlighted existing strengths within the system of care for urban underserved populations, but also many fault lines. When care transitions go poorly during a pandemic, the implications for population health include the risk of higher community transmission, worsening of poverty and other social determinants of health, and increased morbidity and mortality. We sought to examine COVID-19-relevant policies involving governments, health authorities, non-governmental organizations, advocacy groups, and community members at the local, provincial, and national levels that impact continuity of care, especially during transitions between care spaces arising from illness. The specific objectives of this study were to (1) describe health and social COVID-19 response policies for urban underserved populations in three cities (Edmonton, Winnipeg, Toronto); (2) examine how these policies impact continuity of care for urban underserved populations; (3) determine whether and how urban underserved community members were engaged in policy processes; and (4) co-develop policy and operational recommendations for optimizing continuity of care for urban underserved populations during public health crises.

## Methods

### Design

**Fig. 1:**
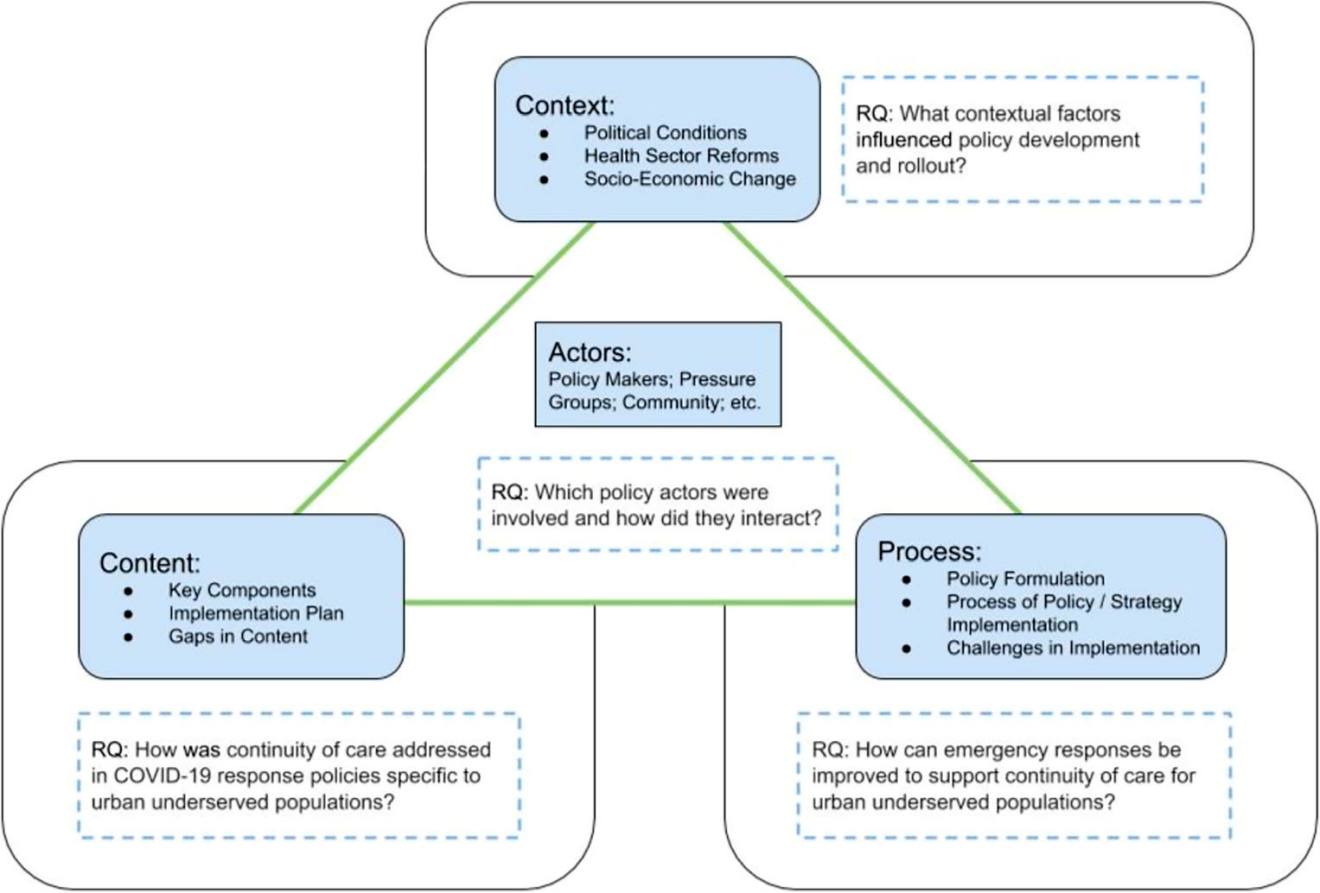
Adapted policy triangle framework. Source: Walt and Gilson (1994)

Case studies are appropriate for in-depth investigation of complex real-world phenomena with numerous intersecting influences; as such, the COVID-19 response is ideal for a comparative case study approach to policy analysis. Health and social policies are developed in the complex interactions between the content of policy, the actors involved, context, and processes. Walt and Gilson’s Policy Triangle framework (Walt & Gilson, 1994) was used to explore the interrelationship and interaction among four main components of policy-making which include actors (individuals, groups, and organizations involved, and their interactions with one another), processes (how policies are formulated and implemented), context (socio-political, cultural, economic, and health and social care setting), and content (the policy’s substance and details such as objectives, decisions made, and implementation plans) within the policy documents from different cities. The policy triangle framework was used to organize and systematically examine how these four components might affect policy decisions on continuity of care for urban underserved populations. Furthermore, the policy triangle is consistent with a definition of policy inclusive of both formal decision-maker directives and broader public interests, ideas, and actions. Whereas most policy analyses focus on policy content, this framework also assesses the context, actors, and processes involved in policy development and implementation. Emphasis on these factors can help promote more effective, informed, and pragmatic policy (Walt & Gilson, 1994) (Fig. 1), which is important in the context of COVID-19 and future emergencies.

### Setting, participants, and partnerships

The case study compared the COVID-19 response from January to December 2020 in urban underserved settings in Edmonton, Winnipeg, and Toronto. These cities provide complementary yet distinct populations, settings, and experiences during the first year of the COVID-19 pandemic (Table 1). Toronto’s urban underserved population is very densely concentrated and multicultural; in contrast, Edmonton has a comparatively large Indigenous population and medium population density, and Winnipeg also has a large Indigenous population (including many northern Manitobans displaced to the urban setting both prior to and during the pandemic) but with a lower population density. With respect to COVID-19 cases during the study period, Toronto saw a higher number of cases earlier in the pandemic, Edmonton witnessed an intermediate but rising number of cases, and Winnipeg experienced its first cases later with fewer first-year cases overall. Public health is primarily administered at the provincial level in Alberta and Manitoba, whereas the municipal level largely directs public health activities in Ontario. All three provinces were governed at the federal level by a centrist party and at the provincial level by a conservative party, but with an ideological mix represented on municipal councils. Last, all three jurisdictions were engaged in active work long term to address continuity of care and care transitions for the general population (Peckham et al., 2018). Policy actors of interest ranged from senior policy makers to front-line service providers, and included the government, health sector (public health, acute care, primary care), and social sector (housing, non-profit organizations).

**Table 1:** Demographics and 2020 COVID-19 cumulative case count for Edmonton, Toronto, and Winnipeg

	Edmonton	Toronto	Winnipeg
Population (2021)	1,010,899^a^	2,794,356^a^	749,607^a^
Number of individuals experiencing homelessness	1651^b^ (2020)	8715^c^ (2018)	1519^d^ (2018)
Number of low-income individuals (2019)	179,140^e^	1,206,880^e^	147,270^e^
Number of Indigenous peoples (2016)	76,205^f^	46,315^f^	92,810^f^
COVID-19 cumulative case count (2020)	44,703^g^	61,675^h^	24,162^i^
Rate of total cases of COVID-19 per 100,000 population (2020)	2287^j^ (Total in Alberta)	1785^j^ (Total in Ontario)	1229^j^ (Total in Manitoba)
Index of remoteness (2016)	0.1332^k^	0^k^	0.2502^k^

*Note*: Data are from Statistics Canada (2022)^a^, Homeward Trust (n.d.)^b^, City of Toronto (2018)^c^, Homeless hub (n.d.)^d^, Statistics Canada (2021)^e^, Statistics Canada (2016a)^f^, Government of Alberta (2022)^g^, City of Toronto (2022)^h^, Government of Manitoba (n.d.)^i^, Government of Canada (2022)^j^, and Statistics Canada (2016b)^k^. Low income counts are for census metropolitan area (CMA), and are larger than counts for each municipality (municipal counts not directly available)

Supporting urban underserved populations during a pandemic requires attention to patient and community engagement, given that the integration of people with lived experience in service planning is associated with increased trust, strengthened relationships between team members, sustained collaboration, and systemic transformation (Jagosh et al., 2012). For traditionally excluded patient populations, this participation can reduce inequity, confer agency, and increase public awareness of issues affecting them (Jürgens, 2005). The COVID-19 crisis has presented significant potential tensions between patient-centred care, patient safety, and infection control. It is unclear to what extent people from urban underserved communities have informed the COVID-19 response. For this reason, the direct participation of people with urban underserved lived experience was prioritized during data collection and interpretation, ensuring that female-identifying, gender non-binary, and Indigenous-identifying individuals were equitably represented among key informants. Moreover, although Indigenous peoples were not the main focus of the study, a larger proportion of urban underserved identify as Indigenous compared to the general population. The team’s community advisory group, over half of whom identify as Indigenous, met periodically during the study to ensure that the study’s design, data collection, interpretation, and dissemination were guided by lived-experience perspectives and priorities. The overall study also received research ethics approval via the University of Alberta, University of Manitoba, and Unity Health Toronto research ethics boards of record.

### Document review

In the first phase of the case study, publicly available written policy documents that address the COVID-19 response specific to urban underserved populations were retrieved and reviewed using a targeted search strategy. Primary data sources included websites of relevant local, regional, and national stakeholders involved in COVID-19 policy and/or health or social policy specific to urban underserved populations. This included Hansard, multi-level government ministries and departments, health authorities, councils, and relevant community, health, and social support agencies. With assistance from a university librarian experienced in searching grey literature and team members familiar with local stakeholders and data sources, internet and database search strings were created and tested using pre-specified COVID-19, urban underserved population, source, and setting terms and modifiers. Using the same search strings, primary source data were complemented by internet searches for media articles to corroborate the timing and content of policy documents and identify relevant policy directions not yet identified in primary data.

January 2020 was set as the start date for collection of Canadian COVID-19 policy content, and active searching continued until December 2020 to capture any policy developments as the pandemic evolved. Policy documents and media articles were considered for inclusion if they addressed the COVID-19 response specific to urban underserved populations. This included policies and media articles for unique settings (e.g. acute care, isolation hotels, addiction treatment facilities) and subpopulations (e.g. women, older adults, Indigenous peoples). Policy documents and media articles were excluded if they were specific to youth and/or children, or if they were not publicly accessible. Policy documents were also excluded if newer, updated versions were retrieved. Two reviewers tested and refined document inclusion and exclusion criteria on initial search results until > 80% agreement was reached. A four-reviewer team subsequently screened and selected documents, ensuring two reviewers screened each document and at least one reviewer for each document was familiar with the city being referenced.

Selected documents were imported into qualitative analysis software (ATLAS.ti). Text sources were analyzed inductively, paying specific attention to document elements that address continuity of care and/or lived experience involvement. Using inductive latent content analysis (Mayan, 2009), two team members reviewed the first 10 primary documents from each study city (for a total of 30 documents) to develop a codebook guided by the four elements of the Policy Triangle framework (context, content, actor, and process) (Walt & Gilson, 1994). Once consensus was reached on the codebook, it was then applied to all remaining documents and refined iteratively, with at least one coding team member familiar with each city being referenced. Codes were then clustered into categories and eventually themes based on the Policy Triangle framework elements. A policy timeline of events was created in tandem with in-depth coding.

### Key informant group interviews

In the next phase, document review findings were triangulated with key informant group interviews. Front-line and lived-experience policy actors from each city with a variety of identities, roles, and perspectives were recruited from each city with assistance from front-line partner intermediaries, focusing on these actors due to their relative under-representation in preliminary review of available policy documents. Front-line partners approached potential participants via existing networks based on their involvement in the planning and/or delivery of COVID-19 response services for the urban underserved, seeking a multidisciplinary and sociodemographically diverse range of informant perspectives. Partners then connected interested individuals to the research team to review study information electronically and provide consent and demographic information prior to focus group sessions. Sessions involving 3–7 participants each were moderated by primarily female-identifying, academic and clinician team members, held using the Zoom virtual platform for public health reasons, and lasted approximately 2 h. Front-line service providers gathered for one session in Edmonton, one in Toronto, and two in Winnipeg, and a final multi-site lived-experience group was also convened. Consenting participants were offered an honorarium of $50 if the session was conducted during personal time to acknowledge their contributions.

Building on the emerging themes from the document review, the semi-structured interview guide assessed how and why policies were developed (or not developed) and whether they were carried out as planned. Because these themes were less prominent in the document analysis, specific questions explored how well policies worked, what would have worked better, how continuity of care was addressed, and how urban underserved populations themselves were engaged. Interviews were audio-recorded and transcribed verbatim, and researchers created field notes.

Again, using an inductive latent content analysis approach and guided by the Policy Triangle framework, research team members applied the document review codebook to interview transcripts. The coding framework was refined for any responses that were not captured within the earlier examined textual data sources. This resulted in the refinement of existing codes but also the development of a modest number of newly arising codes. Following this descriptive analysis, qualitative analysis software was used to perform cross-case comparisons. Major findings from the document review and interviews from each case were compared and synthesized.

## Results

**Fig. 2:**
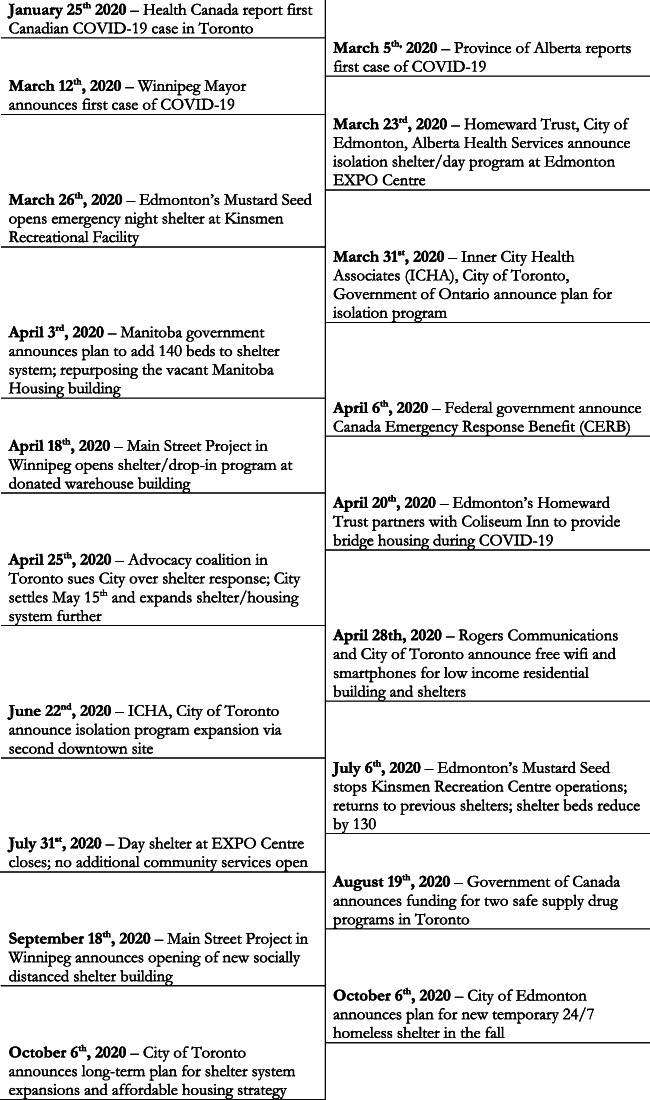
Timeline of key policy events

A total of 237 public-facing local, regional, and national-level policy documents and media articles that spoke to policy decisions published between January and December 2020 were retrieved and analyzed, including 53 government, 51 non-government, and 133 media texts. Five group interview sessions were then held with 22 participants, including 16 front-line practitioners and six lived-experience individuals. Further breakdown of document and key informant sample characteristics are presented in Tables 2 and 3 respectively, and a full list of policy documents by type and jurisdiction is presented in Table 4. A high-level policy event timeline is also presented in Fig. 2. The findings which follow provide a synthesis of the triangulated policy document and key informant interview themes organized according to the four Policy Triangle framework elements, followed by a fifth section on *promising practices* that were highlighted by participants for their positive impact during the pandemic and potential future benefit. Policy *content* and *context* were similar across all three cities, whereas the *actors* and *processes* were more varied. With the exception of digital literacy barriers (a theme unique to interview data), presented themes were present in both document and interview data sources, with the interviews lending additional context and nuance to document-derived themes.

**Table 2:** Policy document characteristics included for review

	Edmonton	Winnipeg	Toronto	National	Total
Government documents	6	5	32	10	53
Health system documents	3	7	16	3	29
Non-government organization documents	6	7	3	6	22
Media documents	39	51	32	11	133
Total	54	70	83	30	237

**Table 3:** Key informant group interview sample characteristics

	Healthcare provider group interview	Informants with lived experience of being underserved group interview
**Total number of informants**	16	6
**Age range**		
20–29	8	0
40–59	8	2
60+	0	3
Not specified	0	1
**Gender**		
Non-binary or not specified	1	2
Female	8	3
Male	7	1
**Ethnicity**		
Indigenous/First Nation/Métis	2	2
South Asian	2	0
Asian	3	0
Caucasian	10	1
Not specified	0	2
**Role**		
Nurse	5	0
Physician	6	0
Pharmacist	1	0
Social worker	2	0
Social service staff	2	1
Peer worker	0	1
Service user	0	2
Not specified	0	2

**Table 4:** Policy document sources

	Organization	Document title	City/national	Document type
1	City of Toronto	Protocol for non-compliance	Toronto	Policy
2	Ontario Ministry of Health	COVID-19 Guidance Consumption and Treatment Services	Toronto	Policy
3	Chief Medical Officer of Health	Operational and outbreak standards for residential addiction treatment service providers	Edmonton	Policy
4	Toronto Public Health	Toronto Public Health Pandemic Plan: A Planning Guide for Housing Service Providers and Shelters	Toronto	Policy
5	Alberta Health Services	Shelter Guidance Preventing, Controlling and Managing COVID-19	Edmonton	Policy
6	Homeward Trust	COVID-19 Updates News	Edmonton	Policy
7	Toronto Public Health	COVID-19 Guidance for Naloxone Kit Distribution	Toronto	Policy
8	Ontario Ministry of Health	Mental Health and Addictions Service Providers in Community Settings	Toronto	Policy
9	Homeward Trust	Coliseum Inn Bridge Housing FAQ final	Edmonton	Policy
10	Alberta Health Services	Opioid Poisoning Response and COVID-19	Edmonton	Policy
11	Region of Peel	Angela’s Place COVID Protocols	Toronto	Policy
12	Toronto Public Health	Responding to overdoses	Toronto	Policy
13	Shelter, Support and Housing Administration	Arranging Non-Emergency Transportation	Toronto	Policy
14	The City of Edmonton	Temporary facility provides homeless people with social distancing opportunities and isolation shelter for COVID-19	Edmonton	Policy
15	Boyle Street Community Services	Our response to COVID-19	Edmonton	Policy
16	Ontario Ministry of Health	Homeless Shelters and COVID-19 Guidance Document	Toronto	Policy
17	Ontario Ministry of Health	MHA Residential Guidance	Toronto	Policy
18	Inner City Health Associates	Homelessness and COVID-19 Testing and Isolation	Toronto	Policy
19	Public Health Ontario	Managing COVID-19 Outbreaks in Congregate Living Settings	Toronto	Policy
20	Inner City Health Associates	COVID-19 Response Mission and Management Principles	Toronto	Policy
21	Inner City Health Associates	ICHA to deliver unique model of care during COVID for people experiencing homelessness in Toronto	Toronto	Policy
22	Region of Peel	Referral Process to the Homeless Response Programs	Toronto	Policy
23	Ontario Ministry of Health	Congregate Living for Vulnerable Populations	Toronto	Policy
24	City of Toronto	COVID19 Drop-in Providers Update	Toronto	Policy
25	City of Toronto	City of Toronto continues to move individuals who experience homelessness from encampments to safe inside spaces	Toronto	Policy
26	Health Commons Solutions Lab	Consultation around COVID-19 recovery sites for people experiencing homelessness	Toronto	Policy
27	Ontario	Action plan: protecting vulnerable Ontarians	Toronto	Policy
28	Region of Peel	Guidance for Homelessness Service Providers	Toronto	Policy
29	Region of Peel	Emergency housing system response	Toronto	Policy
30	Public Health Ontario	Preparedness and Prevention in Congregate Living Settings	Toronto	Policy
31	Toronto Public Health	Guidelines for Harm Reduction Outreach and Community Overdose Response	Toronto	Policy
32	York Public Health	Guidance Document for Service Providers Substance Use and Harm Reduction	Toronto	Policy
33	Shelter, Support and Housing Administration	Shelter, Respite, and Women’s Drop-in Q&A	Toronto	Policy
34	Region of Peel	Shelter Overflow Facilities	Toronto	Policy
35	Region of Peel	Covid-19 Recovery Program	Toronto	Policy
36	York Public Health	Emergency Housing Service Settings	Toronto	Policy
37	Region of Peel	Covid-19 Isolation Program	Toronto	Policy
38	Inner City Health Associates	Isolation Site for Individuals Who Are Homeless	Toronto	Policy
39	The City of Edmonton	City of Edmonton renews State of Local Emergency to continue to protect public safety	Edmonton	Policy
40	The Edmonton Expo Centre	COVID-19	Edmonton	Policy
41	Alberta Government	Alberta Supports Office Closures	Edmonton	Policy
42	The City of Edmonton	City of Edmonton declares State of Local Emergency	Edmonton	Policy
43	The City of Edmonton	City enacts further measures to protect and assist citizens	Edmonton	Policy
44	Alberta Health Services	Harm Reduction and COVID-19	Edmonton	Policy
45	Homeward Trust	COVID-19 Resources	Edmonton	Policy
46	City of Edmonton	Responding to Homelessness in our Communities	Edmonton	Policy
47	Shared Health	COVID-19 Alternative Isolation Accommodation	Winnipeg	Policy
48	Main Street Project	COVID-19 Update (March 24^th^)	Winnipeg	Policy
49	Main Street Project	COVID-19 Update (May 1^st^)	Winnipeg	Policy
50	Government of Manitoba	Information for Shelter Operators	Winnipeg	Policy
51	Government of Manitoba	Public Health Guidelines for Screening Clients of Shelters	Winnipeg	Policy
52	Government of Manitoba	Help stop the spread of COVID-19 infographic	Winnipeg	Policy
53	Aurora Recovery Centre	Aurora’s COVID-19 Action Plan	Winnipeg	Policy
54	Canadian Network for the Health And Housing of People Experiencing Homelessness	Commentary on Health Canada’s Guidance for providers of services for people experiencing homelessness	National	Policy
55	The Canadian Press	Toronto settles suit with homeless advocates over COVID-19 shelter distancing	Toronto	Media
56	CTV	Toronto opens second COVID-19 recovery site for people experiencing homelessness	Toronto	Media
57	Global News	Toronto homelessness advocates sue city over COVID	Toronto	Media
58	Orillia Matters	Pandemic deadly for people suffering from addiction	Toronto	Media
59	Edmonton Journal	Edmonton homeless camp enforcement paused during pandemic	Edmonton	Media
60	The Lawyers	Toronto coalition launches website to help protect people experiencing homelessness from COVID-19	Toronto	Media
61	CBC News	City officials scrambling to add showers and laundry facilities at COVID-19 drop-in centre	Edmonton	Media
62	CTV News	Hinshaw, Kenney defend mats-on-the-floor COVID-19 plan for homeless	Edmonton	Media
63	NEWS	Toronto to distribute $5M to community services helping vulnerable populations	Toronto	Media
64	Toronto Sun	Tent cities highlight homeless crisis	Toronto	Media
65	BlogTO	Toronto’s community centres move out homeless residents as they prepare to reopen	Toronto	Media
66	Government of Canada	How to apply CERB with CRA	National	Policy
67	Government of Canada	Who can apply CERB with CRA	National	Policy
68	CBC	Masks to be mandatory in Toronto’s homeless shelters due to COVID-19	Toronto	Media
69	Global News	City says temporary midtown Toronto homeless shelters to be vacated this week	Toronto	Media
70	BlogTO	Toronto wants to build 3,000 affordable homes because shelters are now too expensive	Toronto	Media
71	Toronto Sun	City keeps public in dark about homeless hotel locations	Toronto	Media
72	Edmonton Journal	Beverly Heights school no longer shortlisted for Edmonton’s new isolation shelter after community pushback	Edmonton	Media
73	The Star	Deadly opioid carfentanil resurfaces in Toronto’s unregulated drug supply	Toronto	Media
74	City of Toronto	Addressing Housing and Homelessness Issues in Toronto through Intergovernmental Partnerships	Toronto	Policy
75	City of Toronto and United Way	Covid-19 Interim Shelter Recovery Strategy Advice from the Homelessness Service System	Toronto	Policy
76	City of Toronto	COVID-19 Seniors & Vulnerable People	Toronto	Policy
77	City of Toronto	Housing and People Action Plan: Responding to the COVID-19 Crisis while Planning for a more Resilient Future	Toronto	Policy
78	Shelter, Support and Housing Administration	Bed Deactivation For Clients Referred To Isolation And Recovery Sites Policy And Procedure	Toronto	Policy
79	Region of Peel	Street Outreach Program	Toronto	Policy
80	Inner City Health Associates	PEACH Resource for Frontline Workers Caring for Clients Experiencing Homelessness in COVID 19	Toronto	Policy
81	Canadian Centre on Substance Use and Addiction	Virtual Care for Mental Health and Substance Use during COVID-19	National	Policy
82	Canadian Association of Emergency Physicians	COVID-19 and Persons Experiencing Homelessness or Vulnerable Housing	National	Policy
83	Health Canada	Government of Canada highlights support for safer drug supply projects in Ontario	Toronto	Policy
84	Centre for Addiction and Mental Health	COVID-19 Opioid Agonist Treatment Guidance	National	Policy
85	Government of Canada	Guidance for providers of services for people experiencing homelessness (in the context of COVID-19)	National	Policy
86	Canadian Research Institute of Substance Misuse	Medications and other clinical approaches to support physical distancing for people who use substances during the COVID-19 pandemic	National	Policy
87	Government of Canada	Helping people who use substances during the COVID-19 pandemic	National	Policy
88	Canadian Research Institute of Substance Misuse	Supporting people who use substances in acute care settings during the COVID-19 pandemic	National	Policy
89	Canadian Alliance to End Homelessness	A Pandemic Response and Recovery Toolkit for Homeless System Leaders in Canada	National	Policy
90	Canadian Research Institute of Substance Misuse	Supporting people who use substances in shelter settings during the COVID-19 pandemic: National Rapid Guidance	National	Policy
91	Canadian Research Institute of Substance Misuse	Telemedicine support for addiction services	National	Policy
92	Toronto Sun	Addiction experts call Throne Speech promises ‘shortcoming’	Toronto	Media
93	CBC	Liberals pledge $1 billion for cities to buy motels, hotels for rapid-housing program	National	Media
94	National Observer	COVID-19 health measures exacerbated opioid crisis: Canada’s top doctor	National	Media
95	CBC	Decriminalization of drugs ‘not a silver bullet’ for overdose crisis, prime minister says	National	Media
96	Globe and Mail	Canada takes step to decriminalize drug possession amid opioid crisis	National	Media
97	CBC	Police chiefs call on Ottawa to decriminalize possession of illicit drugs for personal use	Toronto	Media
98	CTV	Millions of dollars in COVID-19 fines disproportionately hurting Black, Indigenous, marginalized groups report	National	Media
99	Rogers Communications Inc	Rogers family donates $60 million to help most vulnerable Canadians dealing with the economic fallout from the COVID-19 pandemic	National	Media
100	Edmonton Journal	Province needs plan to isolate homeless who fall ill, non-profit says	Edmonton	Media
101	Edmonton Journal	Edmonton declares state of local emergency, free transit and property tax relief	Edmonton	Media
102	CBC News	Iveson demands province protect city’s homeless and broader population from COVID-19	Edmonton	Media
103	Edmonton Journal	City increasing transit security, shuttle service to aid homeless	Edmonton	Media
104	CBC News	Hotels for homeless Edmonton aims to buy ‘surplus’ buildings for winter	Edmonton	Media
105	CBC News	‘A very dangerous situation’: Advocates urge province to change AISH rules due to COVID-19	Edmonton	Media
106	CBC News	Camp Pekiwewin issues new demands, collaborates with service agencies	Edmonton	Media
107	Edmonton Journal	Coliseum Inn activated as 98-unit temporary shelter for homeless residents	Edmonton	Media
108	CBC	Danger increases for homeless Edmontonians since Expo Centre closure, advocates say	Edmonton	Media
109	CBC News	Alberta shelters brace for domestic violence surge linked to COVID-19	Edmonton	Media
110	CTV News	New homeless camp appears as Edmonton works towards a housing solution	Edmonton	Media
111	CTV	Outdoor library services now available through EPL on the Square	Edmonton	Media
112	Edmonton Journal	Edmonton homeless shelters lose 130 beds with reactivation of Kinsmen	Edmonton	Media
113	Edmonton Journal	Edmonton resumes homeless camp removal, focused on camps that pose health, safety risk	Edmonton	Media
114	CBC News	City looks to curb disorder around Expo Centre shelter	Edmonton	Media
115	Edmonton Journal	Outreach workers organize river-valley campout advocating for homeless	Edmonton	Media
116	Global News	Feds pushed on plan to buy vacant properties for affordable housing	Edmonton	Media
117	Edmonton Journal	Steep increase in needles collected on Edmonton public property during COVID-19 pandemic, city data highlights	Edmonton	Media
118	Global News	Opioid-related emergencies in Edmonton more than double	Edmonton	Media
119	City News Toronto	Protestors clash over Toronto homeless housing project amid concerns of safety	Toronto	Media
120	The Star	Today’s coronavirus news: Toronto adds 560 new beds for homeless for winter; de Villa warns outbreak in Toronto could be worse than April; COVID-19 cases increased 40% in Canada over past 7 days	Toronto	Media
121	CTV	Appointment-based COVID-19 testing leaves behind vulnerable people, Ontario doctor says	Toronto	Media
122	Canadian Mental Health Association	Government of Ontario COVID-19 recovery must address mental health and addiction crisis warn experts	Toronto	Policy
123	Globe and Mail	Private clinics allow people to bypass COVID-19 testing line for a fee	National	Media
124	City of Toronto	2020-2021 Winter plan for people experiencing homelessness	Toronto	Policy
125	Canadian Network for the Health And Housing of People Experiencing Homelessness	Briefing and Recommendations: Isolation and Quarantine COVID-19 in the Homelessness Service Sector	National	Policy
126	Shared Health	COVID-19-RAAM-CLINICS	Winnipeg	Policy
127	Manitoba Harm Reduction Network	Open Letter	Winnipeg	Policy
128	Government of Manitoba	Community serving agencies and outreach work	Winnipeg	Policy
129	Government of Manitoba	Outreach guidelines during COVID-19	Winnipeg	Policy
130	Manitoba Harm Reduction Network	COVID-19 Harm Reduction Tips infographic	Winnipeg	Policy
131	End Homelessness Winnipeg	Update on Family Violence	Winnipeg	Policy
132	Addictions Foundation Manitoba	Attention	Winnipeg	Policy
133	End Homelessness Winnipeg	COVID-19 Resources for Winnipeg’s Homeless-Serving Sector - End Homelessness Winnipeg	Winnipeg	Policy
134	Edmonton Journal	Old Strathcona homeless camp relocates to a park up the street after Monday eviction	Edmonton	Media
135	Global News	Edmonton mayor asks province for $17M in annual funding for supportive housing services	Edmonton	Media
136	Global News	Edmonton Convention Centre to be temporarily used to house homeless people	Edmonton	Media
137	Edmonton Journal	Strathcona homeless camp folds tent, citing spike in overdoses	Edmonton	Media
138	Global News	Edmonton hotels show interest in supporting city’s housing needs	Edmonton	Media
139	Global News	Rossdale residents seek solutions amid increase in crime, social disorder	Edmonton	Media
140	CBC	Bridge housing in northeast Edmonton met with mixed feelings	Edmonton	Media
141	CBC News	City approves 4-agency team to run homeless shelter in Edmonton Convention Centre	Edmonton	Media
142	CTV	WINhouse closes both Edmonton shelters after coronavirus outbreak	Edmonton	Media
143	CTV	COVID-19 outbreak reported at Edmonton homeless shelter	Edmonton	Media
144	Calgary Herald	Harm reduction advocates say UCP needs to prioritize opioid crisis	Edmonton	Media
145	CBC News	COVID-19 testing site for Indigenous people to open in Toronto, CBC News	Toronto	Media
146	Government of Ontario	Ontario Expanding Mobile Crisis Services to Respond to Mental Health Emergencies	Toronto	Policy
147	The Canadian Press	Overdoses rise as COVID-19 worsens opioid crisis	National	Media
148	Toronto Sun	Decriminalize simple drug possession, urges T.O. top doc	Toronto	Media
149	Torontocom	‘This is the last door on the road for a lot of people.’ How the pandemic changed Alcoholics Anonymous — possibly forever	Toronto	Media
150	BlogTO	People are saying Toronto’s new homeless shelter looks like a prison	Toronto	Media
151	Edmonton Journal	Homeless shelter maxes out as Rossdale camp closes amid heavy snowfall	Edmonton	Media
152	Edmonton Journal	Rossdale homeless camp cleared by the city, residents encouraged to access 24-7 shelters	Edmonton	Media
153	CBC	Edmonton’s isolation shelter set to expand, convention centre outbreak grows to 22 cases	Edmonton	Media
154	Edmonton Journal	Edmonton Convention Centre shelter COVID-19 outbreak grows to 42 cases, on-site testing to be offered for close contacts	Edmonton	Media
155	CBC News	Conditions at Edmonton Convention Centre shelter unsafe, clients say	Edmonton	Media
156	Toronto Star	Get opioid overdose prevention and harm reduction into Toronto shelters - now	Toronto	Media
157	CBC News	Additional supports coming for communities severely impacted by COVID-19, says Tory	Toronto	Media
158	CTV News	‘Is the LCBO closed?’: What Ontario’s lockdown of Toronto and Peel Region means for retail and other businesses	Toronto	Media
159	City of Toronto	City of Toronto continues to take extraordinary steps to help and protect people experiencing homelessness during COVID-19	Toronto	Media
160	CBC News	People experiencing homelessness safer in tents than shelters during pandemic, Toronto court hears	Toronto	Media
161	Toronto Public Health	Harm Reduction During COVID-19	Toronto	Policy
162	City of Toronto	Anti-Black Racism Analysis Tool for a Radically Equitable COVID-19 Response	Toronto	Policy
163	City of Toronto	COVID-19 Income Support	Toronto	Policy
164	City of Toronto	Enhanced COVID-19 Supports for Targeted Neighbourhoods	Toronto	Policy
165	City of Toronto	COVID-19 Guidance for Emergency Warming Centres	Toronto	Policy
166	Canadian Alliance to End Homelessness	Getting Back to Housing	National	Policy
167	Canadian Alliance to End Homelessness	COVID-19 Home Visits	National	Policy
168	Government of Canada	Who can apply: Canada Recovery Benefit (CRB)	National	Policy
169	Government of Canada	After CERB: Transitioning to new benefits	National	Policy
170	StreetHealth OPS	COVID and Drug Use	National	Policy
171	CBC News	Advocates for unhoused people demand that Toronto stop clearing encampments in parks	Toronto	Media
172	Government of Ontario	Ontario Increasing Mental Health and Addictions Services (Ontario Newsroom)	Toronto	Policy
173	CTV News	Canada launches phone line to prevent overdose deaths	National	Media
174	CBC	Winnipeg’s homeless struggle with physical distancing	Winnipeg	Media
175	CBC	‘An impressive effort’: Hotels, agencies working to provide self-isolation spaces for Manitoba health workers	Winnipeg	Media
176	CBC	‘Why did it take a crisis?’: COVID-19 housing for homeless too little, too late, critic says	Winnipeg	Media
177	CBC	With ridership plummeting, bus shelters become hot spot for injection drug users, Bear Clan says	Winnipeg	Media
178	CBC	‘Wasn’t a blueprint to do it,’ but isolation centre has already welcomed nearly 30 homeless Winnipeggers	Winnipeg	Media
179	CBC	New Siloam Mission centre adds more shelter beds, programming space	Winnipeg	Media
180	Golden West	COVID-19 funding to help 17 Winnipeg programs ending homelessness	Winnipeg	Media
181	Winnipeg Sun	Report shows Winnipeg’s weak points	Winnipeg	Media
182	Winnipeg Sun	Looming winter cold signals action for homeless resource facilities	Winnipeg	Media
183	Winnipeg Sun	COVID pressure test shows cracks in homeless supports	Winnipeg	Media
184	CTV News	New Drug of Choice on Winnipeg Streets, Naloxone Use Sky Rockets	Winnipeg	Media
185	Winnipeg City News	Delay in pandemic-related rise in homelessness gives feds time to prevent it report	Winnipeg	Media
186	CBC	Signs of opioid overdoses surging in Winnipeg during pandemic	Winnipeg	Media
187	CTV News	‘Serious crisis’: Poverty advocate calls for province to enact eviction ban	Winnipeg	Media
188	CBC	Time for homeless encampment near Disraeli Freeway to come down, says Main Street Project	Winnipeg	Media
189	CTV News	Person experiencing homelessness tests positive for COVID-19 in Winnipeg	Winnipeg	Media
190	CBC	Homeless shelters get $760K boost from Manitoba government	Winnipeg	Media
191	CBC	‘Alarming’ signs of pandemic overdose spike emerge in Winnipeg	Winnipeg	Media
192	CBC	Winnipeg homelessness groups create COVID-19 response team to protect high-risk population	Winnipeg	Media
193	CBC	More needs to be done to help homeless during coronavirus outbreak, Winnipeg shelter head says	Winnipeg	Media
194	CTV News	Anti-poverty advocates calling on province to support renters	Winnipeg	Media
195	CBC	Shortages of money and food complicate Morberg House’s pandemic plans	Winnipeg	Media
196	CBC	COVID-19 prompts acceleration of $2-million Main Street Project expansion	Winnipeg	Media
197	APTN News	COVID-19 pandemic putting pressure on women’s shelters	National	Media
198	APTN News	Manitoba women’s shelters preparing for increase in calls as province begins to reopen	Winnipeg	Media
199	APTN News	Unintended consequences as homeless collect emergency benefit, anti-poverty advocates warn	National	Media
200	Winnipeg Sun	COVID-19 wreaking havoc on those experiencing homeless, extreme poverty	Winnipeg	Media
201	CTV News	Inside Winnipeg’s self-isolation centre for the homeless	Winnipeg	Media
202	CBC	As the pandemic’s second wave digs in, Winnipeg’s homeless shelters brace for a bleak winter	Winnipeg	Media
203	News Winnipeg	Addictions Foundation of Manitoba confirms cases of Covid-19	Winnipeg	Media
204	Winnipeg Sun	Nine Winnipeg orgs battling homelessness share $1M in federal funding	Winnipeg	Media
205	CBC	Covid-19 forces Winnipeg’s Main Street Project, Salvation Army to reduce addictions services	Winnipeg	Media
206	CBC	As opioid use spikes during pandemic, consumption sites should be ‘a no brainer’	Winnipeg	Media
207	CBC	Manitoba adding 140 beds for Winnipeg homeless population to self-isolate	Winnipeg	Media
208	Golden West Broadcasting	COVID cases rising in Winnipeg’s homeless population	Winnipeg	Media
209	Shared Health News Release	New 138-bed alternative isolation accommodation site opens in Winnipeg	Winnipeg	Policy
210	Winnipeg Sun	COVID-19 making homelessness problem even worse: Advocate	Winnipeg	Media
211	CTV News	More seeking mental health and addiction support during pandemic	Winnipeg	Media
212	CTV News	‘It’s almost like a double challenge’: Homeless shelters preparing for winter with COVID-19	Winnipeg	Media
213	CBC	Main Street Project gets bulk of $1.5M in funds for Winnipeg homeless shelters	Winnipeg	Media
214	CBC	New Main Street Project shelter will offer ‘more dignified services’ to clients	Winnipeg	Media
215	CTV News	More overdoses, fewer drugs: how the pandemic is changing drug use	Winnipeg	Media
216	CBC	First Nations people with COVID-19 urged to quarantine off reserve in isolation facilities	Winnipeg	Media
217	CMHC	Government of Canada announces support for Rapid Housing in Winnipeg	Winnipeg	Media
218	Winnipeg Sun	Manitoba gov’t enhances access to Naloxone	Winnipeg	Media
219	CBC	Takeout, physically distanced meals: How Manitoba shelters are serving Christmas dinner	Winnipeg	Media
220	CTV News	Temporary restrooms open in Downtown Winnipeg for those experiencing homelessness	Winnipeg	Media
221	CTV News	Winnipeg warming centre to open early this year to help city’s homeless population	Winnipeg	Media
222	CTV News	Manitoba providing $900K to help those going through withdrawal	Winnipeg	Media
223	CTV News	Bus shelters last resort for homeless population	Winnipeg	Media
224	Canadian Mortgage and Housing Corporation	Main Street Shelter opens in Winnipeg	Winnipeg	Media
225	Global News	5 projects to bring 88 new affordable housing units to Winnipeg	Winnipeg	Media
226	Shared Health	AIA Hotel Terms	Winnipeg	Policy
227	CBC	Patrolling streets, building connections: Volunteers reach out to homeless, drug-addicted during pandemic	Winnipeg	Media
228	Make Poverty History Manitoba	Letter to premier	Winnipeg	Media
229	West End	COVID 19 plan	Winnipeg	Policy
230	West End	24hr Safe Space COVID Plan	Winnipeg	Policy
231	CTV News	City of Toronto threatens to remove tiny shelters built to help the homeless, citing safety concerns	Toronto	Media
232	CTV News	Pandemic highlights lack of access to public washrooms during winter months	Toronto	Media
233	CTV News	Leamington family donates $1 million to Toronto-area hospice for homeless community	Toronto	Media
234	CBC	Academics, lawyers, musicians launch public letters urging city to halt encampment evictions	Toronto	Media
235	City of Toronto	City of Toronto announces another round of TO Supports funding for community services to help vulnerable populations	Toronto	Policy
236	City of Toronto	Integrated Prevention and Harm Reduction (iPHARE) initiative	Toronto	Policy
237	City of Toronto	City of Toronto welcomes residents to first modular, supportive housing building	Toronto	Policy

### Policy content

Across all three jurisdictions, the COVID-19 policy response occasionally addressed continuity of care. However, it was not a key consideration for most policy documents—emphasizing instead the control of COVID-19 spread—and a number of policies were developed that could have unintended negative care impacts for urban underserved populations. Strategies recommended to maintain health and social service continuity included using technology to bridge continuity gaps and adjusting service delivery locations and formats (e.g. group to individual). For example, as described in a policy document from the Ontario Ministry of Health:

*It is recognized that much of the support and care that is provided by community-based Mental Health and Addiction service providers may not be deferred. Organizations are encouraged to customize and prioritize service. 8*

These written policy recommendations did not consider the interpersonal nature of support for underserved populations and the importance of face-to-face engagement to promote continuity, which was highlighted by one key informant:

*I would say because more people came for physical follow-up visits in the past, in general I find I have a higher success rate among that population with in-person visits than I do with getting them on the phone. EP2*

For drug poisonings in particular, this loss of contact from new isolation requirements could have devastating consequences:

*People who are used to living in congregate spaces now have own rooms, closed doors, even when you had overdose prevention sites in the hotels people would still understandably want to use in their own rooms and with a toxic drug supply that’s led to, as we’ve seen from coroner’s reports the catastrophic losses that are more than double previous years. TP1*

Moreover, many early government-level policies reflected a lack of familiarity with resource availability on the front lines (e.g. lacking hardware to participate in virtual care) or community members’ personal resources (e.g. lack of family support, internet/phone access, ability to shelter in place) and required subsequent adjustments later in the pandemic. Adjustments included Toronto offering Wi-Fi and cellular supports, Edmonton offering free transportation to COVID-19-specific care spaces, and Winnipeg opening additional winter shelter spaces.

Policies also focused primarily on people experiencing visible homelessness, with minimal attention to the pandemic-related continuity needs of other structurally disadvantaged groups such as precariously or unsafely housed individuals, people experiencing violence, or people who use drugs. Notably absent in any formal policy was specific guidance for women and gender non-binary groups, apart from shelter bed allocations for women and reminders to service providers to collaborate as needed:

*System leaders in homeless services should ensure they are informed of the response being taken in the Violence Against Women sector, and effectively, and as necessary confidentially, communicate that information to services. 89*

Indigenous peoples were more frequently mentioned, with the provision of specific cultural supports in Housing First bridge housing facilities in Edmonton and isolation shelters in Winnipeg, and the creation of a parallel support strategy for Indigenous peoples facing homelessness in Toronto.

Implemented supports were also often temporary despite potential advantages to longer-term care models. In all three cities, for example, documents and key informants verified that live-in isolation facilities with on-site primary care–style supports were implemented for COVID-19–symptomatic people experiencing homelessness:

*But now they might be there for a couple weeks because they’re recovering so it gives people a lot more time to get to know that person and get maybe thinking about it might be good to call their family doctor while they’re here and set up an appointment for the day after they leave and hey, they’re not on [income support], maybe we should get them set up with income. W2P3*

However, most documents stated that these supports would be retracted upon resolution of the pandemic. Many group interview participants in all three jurisdictions worried about the impact of these losses:

*I worry about the outcome of that when that is pulled away. ‘Sorry, we don’t have those services for you anymore and there’s not a pandemic’, right. W1P1*

In summary, policy content was primarily focused on infection control, provided guidance and resources for specific groups (e.g. visible homeless) but not others, offered temporary support without clear post-pandemic transition planning, and infrequently addressed front-line resource scarcity or how to mitigate the risks of social isolation.

### Policy context

The common policy agenda across all jurisdictions’ documents was the need to protect the health of the general population, with the aim of protecting the health of urban underserved populations (homeless populations in particular) being secondary and less immediate in overall agenda setting. Key informants also explained that policies were developed within a context of multiple pre-existing system constraints as well as emerging system strain, and that cities seemed unprepared:

*It’s like my city does not really have a backup plan when something disaster like happens. So, everything is like chaos in the beginning, city just tried to scramble, scramble anything temporary to put all these people, homeless people, people who need a warm place to stay overnight you know. LEP6*

With few pre-existing emergency planning documents addressing emergency-associated health system overload, staff shortages, and socioeconomic changes, strategies were developed de novo to mitigate impending crises:

*Plan for employee absences and prepare by cross-training staff. Resource operations as needed so that the focus can be on essential services. Be creative and flexible in service delivery. 44*

Some policies were also developed to address challenges with pre-existing capacity and accessibility issues such as communal shelter designs. Many social and community services that facilitate access to and continuity of care, such as in-person income support services and drop-in spaces, were markedly restricted. Other services required major expansion, relocation, and/or co-location to adhere to infection control measures. These sudden and sometimes disruptive changes to usual support pathways created intersecting challenges with COVID-19. Group interview participants alluded to even more difficult system navigation than usual:

*A lot of our participants ended up collecting CERB and being cut off assistance and then losing housing so, that was a real difficult thing to navigate and having meetings and advocacy with employment and income assistance to try and continue that relationship. It’s hard enough for our clients to get on assistance, it was just making it more difficult as the whole welfare system shut down and had only one contact number too. W1P2*

Participants also lamented the markedly reduced access to trusted care providers:

*Every few months I have to see my family doctor but since COVID hit, I haven’t seen her since March 2020 and then because of COVID, you don’t see the doctor in person, most of them are virtual you know and at that time I don’t have a phone line to call to talk and so, everything is broke down. LEP6*

In tandem, participants witnessed the loss of access to safer common spaces in which to connect, exacerbating care fragmentation even further through social disconnection and the loss of direct system navigation support:

*With physical distancing measures, we haven’t been able to let people just hang out in our waiting room. For obvious reasons, but I do think that has maybe interfered with being able to locate patients because traditionally you could just put a note on someone’s chart and say okay, they hang out here all the time, they don’t have a phone, but we know they’re going to be just hanging out. EP1*

Thus, the COVID-19 response seemed to occur within an already-strained system of care that was under-prepared for a new public health crisis, compounding pre-existing barriers to access and continuity of care and reducing the available opportunities to provide system navigation and social connection.

### Policy actors

Governments and health system decision-makers were the most common actors in policy development. However, non-governmental organizations (NGOs) and front-line teams often stepped in to develop a response where policy gaps existed. Government documents were primarily guidance documents, whereas NGO documents described fully implemented responses (e.g. Winnipeg Main Street Project’s nine-point priority actions (48); Edmonton coalition of NGOs delineating hours, partners, and services on site at temporary support facility (6)), reflecting the role of these actors in policy development.

Documents also highlighted that relative involvement of different actors varied by jurisdiction. In Edmonton and Toronto, governments and health system decision-makers figured prominently, with municipal governments most directly involved in policy. Community-based NGOs were actively involved in population-specific planning groups, and corporate groups (e.g., hotels for emergency isolation shelter in both settings, Toronto telecommunications involvement to facilitate virtual care) were brought into discussions to facilitate policy implementation. In contrast, the Winnipeg response saw private groups and NGOs leading the policy response and pushing decision-makers for funding and a comprehensive policy framework.

Though there were examples of engagement, very little policy in any of the three cities was informed by or developed in collaboration with community members, or even front-line workers:

*Honestly, I didn’t see shit. Oh, excuse my language. But I didn’t see any of our community members getting consultation about COVID at all. Just all of a sudden COVID hit and then all of a sudden boom, okay here’s this temporary shelter and boom, here’s what’s going on, boom wear your masks, boom get tested just nobody asked. LEP3*

Instead, urban underserved community members and advocates were vocal in the media, where they expressed concerns about such intersecting issues as drug poisonings, income support, and housing, and pushed for policy change. In Edmonton, for example, community members developed an encampment which put pressure on changes to local policy:

*Camp Pekiwewin has issued a new set of demands as organizers partner with inner-city agencies while continuing to pressure governments into lasting changes for Edmonton’s homeless community. 106*

In summary, the relative involvement of different policy actors varied considerably by city. Government and health system actors provided more proactive direction in some cases and more reactive direction in others. Front-line and NGO actors provided the comprehensive details necessary for policy implementation. Community members were seldom directly engaged and instead informed policy through informal means such as the media.

### Policy process

Jurisdictions adopted slightly different approaches to policy development and implementation. In Winnipeg, bottom-up grassroots initiatives began at the NGO level without significant government-level collaboration, as explained by one key informant:

*So, we also came together as different programs, outreach networks, did a lot of stuff by email, online, sharing resources, Facebook was huge, being able to post resources and find foodbanks and getting the word out. W2P2*

These non-governmental actors publicly called for increased government funding and action (e.g. COVID-19 outreach van, alternative isolation accommodations), but community members were not directly engaged.

In Edmonton, the approach was primarily top-down with multilateral collaboration between NGOs, municipal government, and other groups; NGOs adapted rapidly and collaborated to support care continuity. As verified by key informants, urban underserved community members were not involved, but made calls for increased funding and action on intersecting issues—in some cases supporting encampments which prompted action. In Toronto, top-down directives were also common but with similar multilateral collaboration, especially around implementation:

*We [front line clinicians] had given the forewarning [about emerging shelter outbreaks] and said as soon as the first signals of that, they went from being interested in what we were doing in funding some isolation support to ‘Please come to the tables, you have to be in these spaces to actually help coordinate.’ So, I think that would be very unusual, it certainly was for us to be invited to formal ministerial meetings and tables around our emergency systems planning. Usually, homelessness is not part of the acute care spaces. TP1*

Most policies did not demonstrate lived-experience collaboration, with the occasional exception of Toronto outreach teams that produced specific outreach and overdose response guidance:

*This [consultation] process resulted in a series of learnings for future [COVID-19] recovery sites, and insights from those on the ground about how to best provide respectful and dignified care for those in need. 26*

Community members and advocates in Toronto made similar calls for policy change, including filing a lawsuit against the city to uphold COVID-19 mitigation strategies in shelters, sparking major system expansion:

*A fairly large legal challenge... resulted in the legally mandated social distancing in shelters, such as the city had to lease over 20 hotels. So not just that they were doing it for isolation and protection, but they were actually mandated to do that by court injunction. TP1*

Thus, different jurisdictions adopted varying degrees of top-down vs bottom-up policy development approaches. Though collaboration between service providers and higher-level decision-makers eventually emerged, few instances of direct community member consultation occurred, contributing to indirect measures taken to influence policy.

### Promising practices

Across all three jurisdictions, the urgent policy window created by the pandemic required equally quick collaboration and coordination, bringing together a variety of stakeholders more efficiently and effectively than in non-pandemic times, through such structures as emergency advisory committees:

*I see the spirit of collaboration continuing not maybe in the same, gusto perhaps… but I do truly believe for the first time I see hope that that collaboration will continue, and something changed during COVID and I’m really happy to see that. LEP4*

On occasion, efforts were also made to include underrepresented groups. For example, Winnipeg developed a COVID-19 committee that prioritized membership for Indigenous peoples, newcomers, and individuals with disabilities.

Supporting virtual connectivity proved feasible in Toronto despite multiple structural barriers, where a partnership between telecommunication networks, charitable groups, and the municipal government supported the provision of shelters and affordable housing units with free wireless access and cellphones. However, this was a temporary intervention only, and did not address digital literacy or other barriers to virtual care:

*The individuals who didn’t have phones and who didn’t have internet, who didn’t have computers, didn’t have case workers to come over and provide their phones for these virtual appointments or coordinate them, because you obviously need someone to call in and book these appointments and if you don’t have a phone, you can’t do that. TP2*

Edmonton chose to co-locate multiple health and social supports (e.g. housing intake, nursing care, isolation beds, supervised drug consumption) within expanded physical spaces:

*That’s what happened here in Edmonton once COVID hit. We had a temporary shelter at the Edmonton Convention Centre, and we had all supports there. LEP3*

Though intended to reduce the risk of viral transmission in smaller agency spaces, this also led to improved coordination between different services. This was paired with free public transportation to promote uptake as new supports were not within walking distance of the most underserved neighbourhoods.

## Discussion

Although some promising strategies have been described to help maintain continuity during care transitions, the results of this policy case study analysis underscore a fragmented care system for urban underserved groups in Canada, echoing the emerging literature on inequities during COVID-19 for structurally vulnerable populations (Wojtak et al., 2020). Documents and key informant interviews especially highlighted pre-existing system capacity and resource challenges. However, the findings also suggest that COVID-19 has presented a unique policy window with opportunity to improve how the health systems support transitions in care for urban underserved populations. Though there were variable policy actor roles and response processes in each city, government and health system decision-makers typically provided high-level guidance and funding, whereas front-line NGOs and other groups collaborated to operationalize and implement a number of innovative support strategies. Ideally, both high-level and front-line policy actors should partner a priori to inform each other’s roles and actions, and adapt to a rapidly evolving context more effectively. These partnerships would benefit from sustained post-pandemic collaboration to review promising innovations emerging from the COVID-19 response and determine how to maintain these supports to improve urban underserved care transitions over the longer term (Heimer et al., 2020; Wenger et al., 2021). Public health must play an active role in this partnered work given the inequities faced by urban underserved populations, the impact on community health, and the potential for upstream prevention.

With few exceptions, people from urban underserved communities were not included in developing policy around the COVID-19 response. This finding is echoed by other COVID-19 research demonstrating a relative lack of patient engagement (Wojtak et al., 2020). A lack of meaningful engagement risks compounding the inequities faced by urban underserved populations such as more frequent COVID-19 acquisition, higher severity, exacerbation of intersecting social issues, and less access to related supports. The sudden imposition of service restrictions, paired with the temporary nature of pandemic supports with no clear plan forward to sustain them (Kaur et al., 2021), may further erode urban underserved communities’ trust in health and social systems. Conversely, as evidenced in this study and elsewhere in the literature, a collaborative and community-centred policy environment can mitigate many of these concerns (Morgan et al., 2021; Heimer et al., 2020). There is a clear need for an equity and justice lens in future emergency responses and policy development around care transitions, which would benefit greatly from a co-design approach with people with lived experience (Sayani et al., 2021).

The COVID-19 response demonstrated key areas of opportunity for policy and decision-makers to support transitions in care for urban underserved populations over the longer term. First, connectivity solutions could support virtual care for under-resourced individuals and organizations (Kaur et al., 2021; Ghidei et al., 2022). As these communication tools may be new for some service recipients, their utility will depend on ongoing front-line support during adoption of virtual options. Second, connecting people to bridge- and long-term housing supports continuity of care, in particular when interdisciplinary teams are integrated into bridge facilities and able to support transitions to more permanent housing. Third, the pandemic has underscored an urgent need to address Canada’s ongoing drug poisoning syndemic and has forced service providers to reconsider traditional approaches to substance use management (Heimer et al., 2020; Wenger et al., 2021). More widely available harm reduction interventions, especially when paired with other supports such as shelters and healthcare settings, may encourage connection to essential health and social services while reducing the risk of death. Last, the co-location of multiple health and social services and removal of structural barriers to access (e.g. transportation) can promote more integrated care overall (Kaur et al., 2021).

### Limitations

This comparative case study comes with several limitations. First, it examined a narrow policy window; not all policy decisions were public-facing or easily retrievable during the early waves of the pandemic. The key informant group interviews were in part designed to capture less well-documented policy and its implementation and reduce the likelihood that major decisions were missed. Another limitation is the use of virtual group interview methods, in which some participants may have experienced tacit digital divides to their full participation despite the offer of connectivity support from the research team. Further, the relatively conservative political landscapes in each province studied influenced the policy context and may potentially limit the applicability of findings to other conservative-leaning jurisdictions rather than more broadly. Last, a full intersectional analysis was beyond the scope of the study; policy documents contained little to no information specific to women, gender-diverse, and Indigenous groups, which restricted the scope of the analysis. The findings herein should be interpreted with caution for specific urban underserved subpopulations, and highlight the need for an intersectional approach to related policy development in the future.

## Conclusion

The COVID-19 pandemic has had a disproportionately negative impact on urban underserved populations. Service constraints, time-delimited supports, intersecting crises, and minimal lived-experience consultation amplified the pre-existing system inequities faced by this population. However, the COVID-19 response has also demonstrated the feasibility of multi-stakeholder collaboration and support. Ongoing partnership in the form of government and decision-maker resources, front-line innovation, and lived-experience involvement is needed for urban underserved populations experiencing care transitions during COVID-19 and beyond.

## Contributions to knowledge

What does this study add to existing knowledge?

• COVID-19 policy responses for Canadian urban underserved populations were largely reactive and temporary, compounding an already inequitable system of care.

• Community members were rarely involved; however, a number of community-based initiatives were developed in response to policy gaps.

• Promising practices emerged that should be considered for longer-term use, including examples of new multi-level and multi-sector collaboration, virtual connectivity supports, and collocation of services.

What are the key implications to public health interventions, practice, or policy?

• Given the inequities faced by urban underserved populations, public health professionals should apply an equity and justice lens in future emergency responses, in direct and timely partnership with people with lived experience and other policy stakeholders.
